# Reversible Effects of Integrase Inhibitors on Newly Differentiated Adipocytes

**DOI:** 10.3390/v18010149

**Published:** 2026-01-22

**Authors:** Richard Taylor Pickering, Archana Asundi, Alex Olson, Katie Soden, Nina H. Lin

**Affiliations:** 1 Chobanian and Avedisian School of Medicine, Boston University, Boston, MA 02118, USA; 2 Boston Medical Center, Boston, MA 02118, USA

**Keywords:** integrase inhibitors, adipocytes, dolutegravir, darunavir, HIV

## Abstract

Weight gain has been associated with integrase strand transfer inhibitors (INSTIs) in real-world studies; however, the causality of this relationship is unclear. Thus, we examined the effects of the INSTI, Dolutegravir (DTG), on human adipose cells in vitro and the reversibility of these effects by switching to a protease inhibitor, Darunavir (DRV). We established cultures of human adipose stem cells (ASCs) and newly differentiated adipocytes from individuals without HIV. For adipocytes, cells were exposed to DTG or DRV for 7 days, after which cells were maintained or switched to another ART. Experiments examining ASCs and the effects on adipogenesis initiated exposure during proliferation and continued throughout differentiation. Adipogenic outcomes included triglyceride content, gene expression, and adipokine secretion. Metabolic outcomes included lactate production, lipolysis, and oxygen consumption rates. DTG suppressed the secretion of adiponectin and leptin, and this was reversed following the switch to DRV in adipocytes without the altered expression of adipogenic genes. DTG exposure increased markers of endoplasmic reticulum stress, elevated lactate production, and suppressed oxygen consumption in ASCs. Exposure to DTG during differentiation lowered triglyceride accumulation and adiponectin secretion without altering the expression of adipogenic markers. Thus, DTG exposure resulted in changes in adipocyte function consistent with the progression of metabolically adverse phenotypes, and these effects were reversible.

## 1. Introduction

Among people with HIV (PWH), there is a growing prevalence of obesity, which may lead to enhanced risk of diseases linked to metabolically unhealthy obesity (MUO), such as cardiovascular disease and diabetes. While MUO in non-HIV populations is well understood, the impact of antiretroviral therapies (ARTs) in PWH is crucial to understanding the phenotype of adipose tissue in this population.

Integrase strand transfer inhibitors (INSTIs), particularly dolutegravir (DTG), have been associated with approximately a 2 kg increase in weight, corresponding to a 0.5–1 kg/m^2^ increase in the body mass index among PWH switching to a new regimen containing an INSTI. In the general population, excess weight is a risk factor for several cardiometabolic comorbidities, including type 2 diabetes mellitus [1]. However, the precise relationships between INSTIs, weight gain, and metabolic risk that have been observed in cohort studies are not well understood. An increase in insulin resistance related to INSTI-emergent weight gain has been described in some cohorts but not all [2,3,4,5]. A strong signal of increased insulin resistance has been linked to switching off efavirenz (EFV)-containing regimens, particularly among slow metabolizers of this non-nucleoside reverse transcriptase inhibitor [6], which is thought in and of itself to be weight suppressive [7], though the role of INSTIs in this effect is not clear.

Prior in vitro studies suggest that some INSTIs directly alter adipogenesis and promote fibrosis through the modulation of genes that control collagen deposition and adipocyte differentiation [8,9], similar to observations in MUO [10,11]. The degree of adipocyte differentiation is strongly linked to metabolic homeostasis, with low adipogenesis being linked to higher hemoglobin A1C [12]. While early generations of ART had dramatic effects on adipose tissue distribution due to mitochondrial toxicity that led to lipodystrophy from the wasting of peripheral depots, modern regimens have been associated with more subtle changes that are less precisely defined. These include those related to chronic adipose tissue dysfunction from generalized weight gain, impaired adipogenesis, and increased fibrosis [13]. Thus, understanding these mechanisms at a cellular level will provide critical insight into how newer generations of ART may impact aspects of adipose tissue biology related to the development of MUO.

Human adipose stem cells (ASCs) have been used to model many aspects of adipocyte and preadipocyte biology due to their relative ease of growth and differentiation, metabolic parity with freshly isolated adipocytes, and physiological “memory” from who and where they were isolated [14]. They permit the mechanistic study of both drugs, genes, and physiological factors that may impact the metabolic health of adipocytes.

In this study, we utilize this model of adipogenesis to understand the cellular effects of DTG on key aspects of adipocyte biology and examine the extent to which these effects are reversible via switching to a different class of ART—the protease inhibitor darunavir—not associated with major fluctuations in weight [15].

## 2. Materials and Methods

### 2.1. Culture and Exposure of Adipose Stem Cells

Adipose stem cells represent a population of precursors that can become adipocytes and secrete a host of pro-inflammatory and profibrotic factors. Frozen stocks of human abdominal subcutaneous adipose stem cells (ASCs) were grown to confluence on 12-well plates in alpha-MEM media containing 10% FBS. Following confluence, ASCs were exposed to DTG (3.1 µg/mL) [16] or Darunavir (DRV) (11.8 nM) [17] for 2 days, after which supernatant, cell lysates, and RNA were collected. These concentrations were used across experiments and were based on Cmax measurements from pharmacodynamic studies.

### 2.2. Generation of Newly Differentiated Adipocytes

Newly differentiated adipocytes closely mimic the key characteristics of adipocytes isolated from biopsies. Thus, experiments examining the responses of mature adipocytes utilized newly differentiated adipocytes under a maximal differentiation protocol described previously [14]. Briefly, ASCs were grown to confluence on 12-well plates in media containing 10% FBS, then treated for 7 days with complete differentiation media (CDM) containing insulin, dexamethasone, Isobutylmethylxanthine, rosiglitazone, and other factors. After 7 days, cells were switched to lipogenic maintenance media. For experiments examining the effect of ART on adipogenesis, exposure to ART was initiated alongside CDM. For experiments examining the effect of ART on newly differentiated adipocytes, exposure to ART was initiated alongside the switch to maintenance media. Cells were collected in either cell lysis buffer (Cell signaling, Danvers, MA, USA) or QIAzol™ (Qiagen, Germantown, MD, USA) for DNA and TG determination or RNA isolation, respectively.

### 2.3. Measurement of Adipokine Secretion

Supernatants from the last 24 h of culture were collected and assessed for adiponectin, leptin, IL-6, and MCP-1 using commercially available ELISAs (R&D Systems, Minneapolis, MN, USA, Table S1). Media content was normalized to total cellular DNA, as newly differentiated adipocytes are post-mitotic.

### 2.4. Assessment of Cellular Oxygen Consumption

Preadipocytes were grown to confluence and exposed to ART for 2 days on 96-well Seahorse plates. On the day of the experiment, cells were switched to seahorse media containing DMSO, DTG, or DRV for 2 h, then subjected to a mitochondrial stress test (Agilent Technologies, Santa Clara, CA, USA).

### 2.5. Isolation of RNA and cDNA Synthesis

Cells were collected in QIAzol™, and RNA was extracted using the phenol-chloroform method, with RNA quality and quantity assessed using Nanodrop (ThermoFisher, Waltham, MA, USA). Libraries of cDNA were synthesized using the High-Capacity cDNA Reverse Transcription Kit (Applied Biosystems, Waltham, MA, USA), with 1 μg of RNA input. Completed reactions were diluted 1:10 for use in RT-qPCR.

### 2.6. Measurement of mRNA Expression

Genes of interest were measured using a QuantStudio™ 3 Real-Time PCR system and Taq-Man Probes (Applied Biosystems, Waltham, MA, USA). Fold expression was calculated using the 2^−ΔCT^ method with PPIA serving as the housekeeping gene. Specific probes can be found in Table S2.

### 2.7. Assessment of Impact on Adipogenesis

Following the synthesis of cDNA libraries from isolated RNA, the expression of mRNAs indicative of late adipogenesis (PPARγ, LPL, and FABP4), as well as triglyceride accumulation (see below), were used to assess the degree of differentiation.

### 2.8. Measurement of Basal Lipolysis and Triglyceride Content

Free glycerol was assessed as a surrogate measure for lipolysis. Briefly, 24 h supernatants from the final day of culture were collected, and glycerol was completely converted to Dihydroxyacetone phosphate using Glycerol Kinase and Glycerol-phosphate Dehydrogenase in the presence of hydrazine hydrate. NADH was measured using excitation-emission spectra and compared to a standard curve. For the assessment of triglyceride content, cells collected in non-SDS-containing cell-lysis buffer were pre-treated with a lipase mixture for 1 h (Sigma-Aldrich, St. Louis, MO, USA), after which free glycerol was measured.

### 2.9. Statistical Analysis

Data are presented as mean ± standard deviation unless otherwise noted. Paired samples are indicated by consistent shapes between bars and represent different ASC donors or the same donor from different stocks thawed on different days. Non-parametric data were first natural log-transformed prior to statistical testing. Differences between means were assessed using 1-way repeated-measures ANOVAs and Tukey’s post-tests. For experiments with missing values for specific conditions, mixed-effects models were utilized in place of 1-way repeated-measures ANOVAs. Statistical analysis was performed using GraphPad Prism 8 (GraphPad Software, Boston, MA, USA).

## 3. Results

### 3.1. ART Exposure to Adipose Stem Cells Did Not Alter Fibrotic or Inflammatory Phenotype

Adipose stem cells are important contributors to both inflammatory and fibrotic characteristics of adipose tissue, and dysregulation in these pathways can contribute to the development of dysfunctional adipose tissue. Thus, to examine the impact of ART on ASCs, we treated confluent cells for 2 days and examined changes in fibrotic gene expression and inflammatory marker secretion. Exposure to DTG or DRV for this period did not alter expression of fibrotic markers (alpha actin [ACTA2], inhibin beta A [INHBA], and tissue inhibitor of metalloproteinase-1 [TIMP1]), compared to the control (*p* > 0.05). Further, neither drug altered the secretion of the pro-inflammatory adipokines MCP-1 or IL-6 (Figure S1) compared to the control.

### 3.2. ART Exposure in Newly Differentiated Adipocytes Does Not Alter Markers of Adipocyte Quality

**Figure 1:**
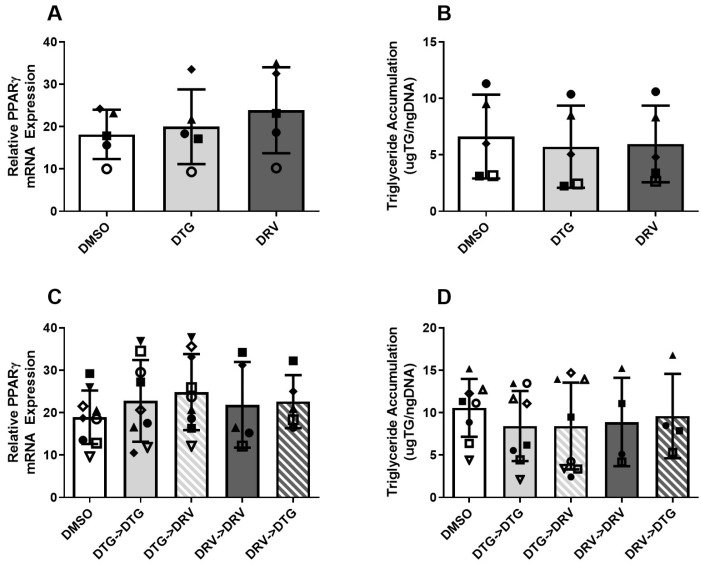
Exposure to Dolutegravir or Darunavir did not alter adipocyte markers in newly differentiated adipocytes. mRNA expression of PPARγ (**A**,**C**) and triglyceride accumulation (**B**,**D**), late markers of adipogenesis, following 7 ((**A**,**B**), *n* = 5) or 14 ((**C**,**D**), *n* = 4–9) days of exposure to ARTs during maintenance; “->” indicates the switch or maintenance on day 7 to the indicated drug. Matched shapes indicate paired experiments. DMSO—Dimethyl Sulfoxide, DTG—Dolutegravir (3.1 µg/mL), DRV—Darunavir (11.8 nM). All legends with these shapes indicate that “Matched shapes indicate paired experiments” and in the main text under “Statistical analysis” the statement “Paired samples are indicated by consistent shapes between bars and represent different ASC donors or the same donor from different stocks thawed on different days” can be found.

Mature adipocytes are responsible for many important biological functions, including the storage of triglyceride, glucose uptake, and the secretion of adipokines. Under certain stimuli, they can undergo dedifferentiation [18], indicated by the decreased expression of key adipogenic genes (e.g., PPARγ) and reduced triglyceride content. This regression limits their ability to carry out the functions mentioned above. Thus, to assess whether DTG has detrimental effects on mature adipocytes, cells were exposed to different ARTs following 7 days of induction. Neither DTG nor DRV had significant effects on the expression of the master regulator of adipogenesis, PPARγ, or triglyceride accumulation after 7 days of treatment (Figure 1A,B). Further, this lack of an effect was consistent after 7 more days of treatment and was not influenced by switching between ARTs (Figure 1C,D). The expression of other late markers of adipogenesis, FABP4 and ADIPOQ, was similarly unaffected (Figure S2).

### 3.3. Exposure to Dolutegravir Suppressed Secretion of Adiponectin and Leptin in a Reversible Manner

**Figure 2:**
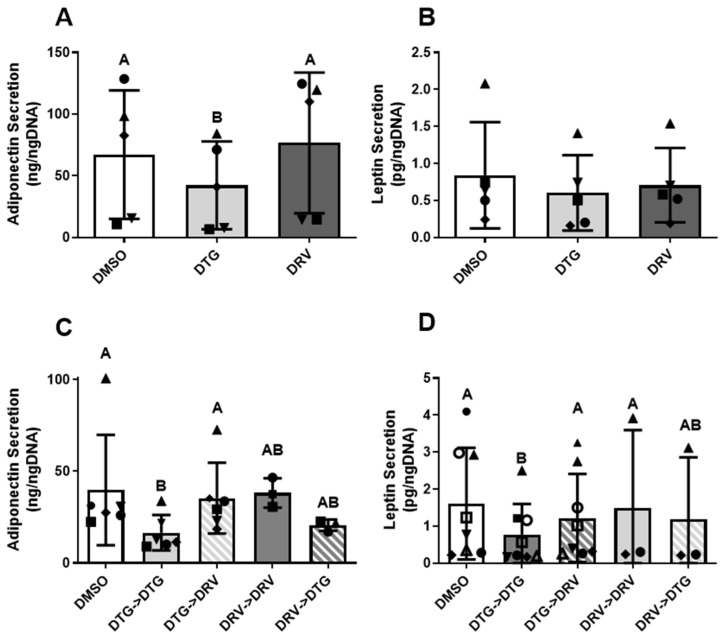
Exposure to dolutegravir decreased secretion of Adiponectin and leptin, reversed by the switch to darunavir. Secretion of Adiponectin (**A**,**C**), *n* = 3–6) and Leptin (**B**,**D**), *n* = 3–8) after 7 (**A**,**B**) and 14 (**C**,**D**) days of exposure to Dolutegravir (DTG, 3.1 μg/mL) or Darunavir (DRV, 11.7 nM) in newly differentiated adipocytes. “->” indicates a switch or maintenance on day 7 to the indicated drug. Matched shapes indicate paired experiments. Different letters signify significant (*p* < 0.05) differences between groups assessed by 1-way repeated-measures ANOVA of log-transformed data; bars with the same letter are not statistically significantly different. DMSO—Dimethyl Sulfoxide. All legends with these shapes indicate that “Matched shapes indicate paired experiments” and in the main text under “Statistical analysis” the statement “Paired samples are indicated by consistent shapes between bars and represent different ASC donors or the same donor from different stocks thawed on different days” can be found.

Adipocytes are a source of cytokines known to influence metabolic and inflammatory processes, and alterations in these molecules can have systemic impacts. We observed that cells exposed to DTG for 7 days with detectable levels of adiponectin had lower secretion of adiponectin compared to cells exposed to DRV (Figure 2A, 42.1 ± 35.6 vs. 76.6 ± 57.1 ng/mL/ngDNA, *p* < 0.01) and compared to DMSO (67.1 ± 52.0 ng/mL/ngDNA, *p* = 0.05). After 14 days, cells exposed to DTG secreted significantly less adiponectin than the control or DRV (Figure 2C, 16.6 ± 9.7 vs. 39.8 ± 30.1 and 38.3 ± 8.0, *n* = 3–6). Further, cells switched from DTG to DRV at day 14 of differentiation saw a return to control levels of adiponectin after 7 days (35.4 ± 19.4 vs. 39.8 ± 30.1, *p* > 0.05 *n* = 6). While it did not reach statistical significance, cells switched from DRV to DTG tended to have lower secretion of adiponectin compared to the control or DRV (20.6 ± 3.1 vs. 39.8 ± 30.1 and 38.3 ± 8.0, *n* = 3–6). The secretion of leptin followed similar patterns, with DTG suppressing secretion compared to the control (0.75 ± 0.77 vs. 1.56 ± 1.42 ng/mL/ngDNA, *p* < 0.05, *n* = 3–8) and switching to DRV reversing this suppression, though not completely (1.17 ± 1.12, *p* > 0.05). In contrast, the inflammatory cytokines IL-6 and MCP-1 were unaffected by either ART (Figure S3).

### 3.4. Dolutegravir and Darunavir May Increase Markers of Endoplasmic Reticulum Stress

**Figure 3:**
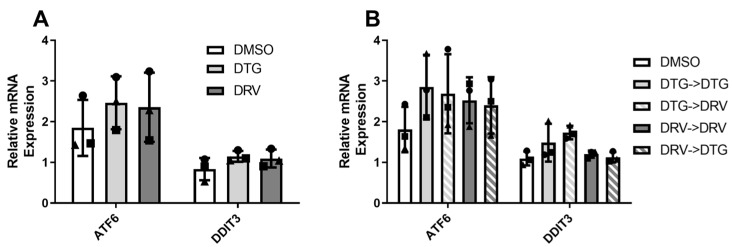
Dolutegravir may increase the expression of markers of ER stress. Expression of ATF6 and DDIT3 mRNAs following 7 (**A**) or 14 (**B**) days of exposure to dolutegravir (DTG, 3.1 μg/mL) or darunavir (DRV, 11.7 nM); “->” indicates the switch or maintenance on day 7 to the indicated drug. Matched shapes indicate paired experiments. DMSO—Dimethyl Sulfoxide, ATF6—Activating Transcription Factor 6; DDIT3—DNA Damage Inducible Transcript 3. All legends with these shapes indicate that “Matched shapes indicate paired experiments” and in the main text under “Statistical analysis” the statement “Paired samples are indicated by consistent shapes between bars and represent different ASC donors or the same donor from different stocks thawed on different days” can be found.

Endoplasmic reticulum stress has pleotropic effects on adipocyte biology, including, but not limited to, reduced adipokine secretion, mitochondrial stress, and the induction of apoptosis [19]; thus, we sought to examine the impact of ART on two ER stress-inducible mRNAs. Figure 3 shows that after 7 days of exposure, both DDIT3 and ATF6 tended to be increased by DTG (ATF6, 2.4 ± 0.7 vs. 1.8 ± 0.7, *p* = 0.11). After 14 days of exposure, both DTG and DRV tended to increase the expression of ATF6 compared to DMSO (2.9 ± 0.8 vs. 2.5 ± 0.6 vs. 1.8 ± 0.6, *p* > 0.1, *n* = 3). Interestingly, switching seemed to exacerbate these effects in either condition compared to the DMSO control (DTG->DRV, 2.7 ± 1.0, *p* < 0.05; DRV->DTG, 2.4 ± 0.7, *p* = 0.1).

### 3.5. Dolutegravir Increased Lactate Production in Adipose Precursor Cells and Newly Differentiated Adipocytes

**Figure 4:**
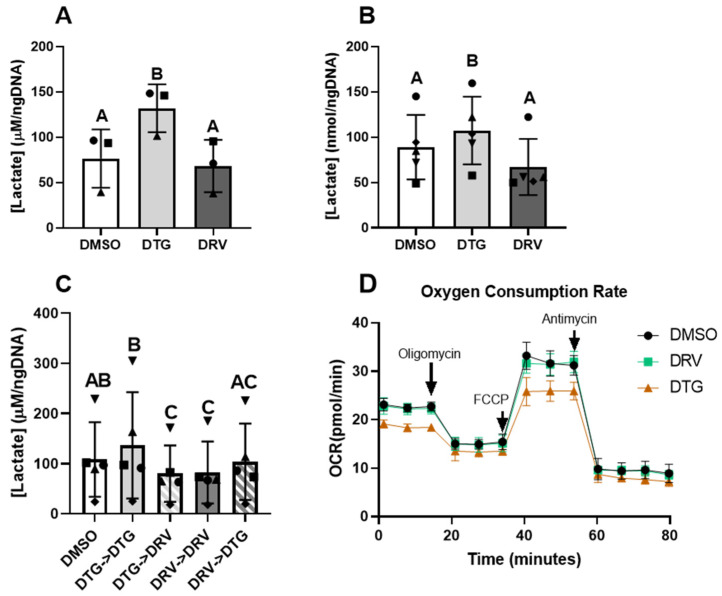
Exposure to dolutegravir may impact mitochondrial function in adipose stem cells and newly differentiated adipocytes. Lactate content in media from adipose stem cells ((**A**), *n* = 3) and newly differentiated adipocytes exposed for 7 days ((**B**), *n* = 5) or 14 days ((**C**), *n* = 5) to dolutegravir (DTG, 3.1 μg/mL) or darunavir (DRV, 11.7 nM). “->” indicates the switch or maintenance on day 7 to the indicated drug Oxygen consumption rates of preadipocytes exposed to DTG and DRV for 2 days ((**D**), *n* = 3). Matched shapes indicate paired experiments. Different letters indicate statistically significant differences between groups (*p* < 0.05) via 1-way repeated-measures ANOVA of log-transformed data; bars with the same letter are not statistically significantly different. DMSO—Dimethyl Sulfoxide. All legends with these shapes indicate that “Matched shapes indicate paired experiments” and in the main text under “Statistical analysis” the statement “Paired samples are indicated by consistent shapes between bars and represent different ASC donors or the same donor from different stocks thawed on different days” can be found.

Early observations of adipocytes exposed to DTG indicated increased acidification of the media via yellowing of the indicator phenol red. Acidification may indicate an excessive reliance on anaerobic respiration. To examine this, we measured lactate content in the media of these cells. Lactate content in the media from adipose stem cells treated for 2 days was significantly higher in those exposed to DTG compared to control or DRV (132 ± 26 vs. 76 ± 32 vs. 68 ± 29 μM/ngDNA, *p* < 0.05, Figure 4A). Further, in mature adipocytes after 7 days of exposure, those exposed to DTG had significantly greater concentrations of lactate (107 ± 37 vs. 89 ± 36 vs. 67 ± 31, *p* < 0.05, Figure 4B). After 14 days of exposure, Lactate was lower in those cells exposed to DRV, and those switched from DTG to DRV after 7 days compared to control and those cells maintained in DTG (DRV->DRV, 82 ± 61; DTG->DRV, 80 ± 56; DMSO, 108 ± 74; DTG->DTG, 137 ± 106, *p* < 0.05) (Figure 4C). Interestingly, cells maintained in DTG for 14 days did not have significantly greater lactate, compared to the control. This increase in lactate at earlier time points may indicate an increased reliance on anaerobic glycolysis and lower mitochondrial oxygen consumption. To examine this, we utilized Seahorse to examine mitochondrial oxygen consumption rates in adipose stem cells exposed to DTG and DRV. We found that cells exposed to DTG had small but significant (10%, *p* < 0.05) reductions in basal and maximal oxygen consumption rates compared to the control (Figure 4D).

### 3.6. Dolutegravir Suppressed Lipid Accumulation in Cells Treated During Differentiation

**Figure 5:**
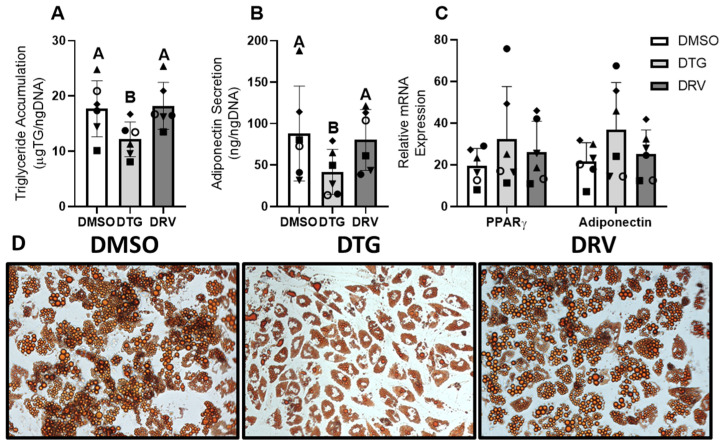
Dolutegravir may suppress differentiation of adipose stem cells. Triglyceride accumulation (**A**), Adiponectin secretion (**B**), and mRNA expression (**C**) of late adipogenic genes in newly differentiated adipocytes exposed to dolutegravir (DTG, 3.1 μg/mL) or Darunavir (DRV, 11.7 μM) throughout adipogenesis and maintenance (*n* = 6). Representative images of oil red O staining on day 14 of exposure (**D**). Matched shapes indicate paired experiments. Different letters indicate statistically significant differences between groups (*p* < 0.05) via 1-way repeated-measures ANOVA of log-transformed data; bars with the same letter are not statistically significantly different. DMSO—Dimethyl Sulfoxide. All legends with these shapes indicate that “Matched shapes indicate paired experiments” and in the main text under “Statistical analysis” the statement “Paired samples are indicated by consistent shapes between bars and represent different ASC donors or the same donor from different stocks thawed on different days” can be found.

The ability of adipose precursor cells to become mature adipocytes is an important factor in the development of metabolic disease, and lower adipogenesis is associated with HbA1c levels and other metabolic abnormalities [12]. Thus, to examine this crucial role of ASCs, we altered the exposure regimen to coincide with this process to examine the impact of ART on adipogenesis. The exposure of adipose precursor cells to DTG beginning at the initiation of differentiation suppressed triglyceride accumulation (12.2 ± 3.1 vs. 17.7 ± 5.1 μgTG/ngDNA, *p* < 0.05, *n* = 6) and the secretion of adiponectin (41.7 ± 27.2 vs. 88.1 ± 57.2 ng/ngDNA, *p* < 0.05, *n* = 6) (Figure 5A,B), but this was not observed with DRV (18.2 ± 4.2 μgTG/ngDNA; 80.4 ± 36.8 ng/ngDNA, *p* > 0.05) (Figure 5C). This is reflected in lipid staining with oil red O (Figure 5D). With these changes, we expected the mRNA expression of late adipogenic markers to be substantially decreased; however, the expression of both PPARγ and adiponectin was paradoxically increased for cells exposed to DTG compared to the control, though this did not reach statistical significance.

## 4. Discussion

We examined the effects of two commonly used antiretroviral medications on different stages of adipocyte development. DTG, but not DRV, resulted in decreased adipogenesis as evidenced by decreased TG accumulation and decreased adipokine secretion. Additionally, DTG increased lactate production in newly differentiated adipocytes with evidence of ER stress. Notably, these phenotypes were reversible when cells were switched to DRV. Elevated lactate production was accompanied by a decrease in mitochondrial oxygen consumption rates in adipose stem cell populations, with no change in inflammatory or fibrotic markers. These changes induced by DTG are consistent with phenotypes seen in the progression of metabolically unhealthy obesity, which can lead to the development of cardiovascular disease, metabolic dysfunction-associated steatohepatitis, and diabetes, diseases overrepresented in PWH [20].

These data add to the growing number of studies examining mechanistic insights into excess weight gain associated with INSTIs in PWH. Our data largely align with prior studies showing that exposure to various integrase inhibitors negatively suppresses adipokine secretion from adipocytes [9,21,22]. To our knowledge, our studies are the first to demonstrate that these effects on adipokine secretion are reversible at the cellular level (Figure 2). Our observations suggest INSTI-mediated adipocyte dysfunction may be reversed by switching to a non-INSTI-containing regimen. This is notable as changes in adipose tissue that affect metabolic health may occur in the absence of clinically apparent weight changes. Therefore, the lack of weight change observed in clinical studies switching from INSTIs to non-INSTI regimens may not be a sensitive enough marker for changes in metabolic function [15,23,24]. Further, our data are consistent with other studies showing little to no effect of DRV on adipocyte functions [25,26].

While DTG led to a decrease in the secretion of both adiponectin and leptin in mature adipocytes, other adipokines (i.e., IL-6 and MCP1) were unaffected by either DTG or DRV (Figure 2 and Figure S3), suggesting that fully differentiated cells were not undergoing profibrotic remodeling or activating inflammatory pathways characteristic of metabolically unhealthy obesity [27]. This is somewhat in contrast with previous reports showing that DTG exposure increased the production of collagen 6 and fibronectin from preadipocytes, newly differentiated adipocytes, and [9] beige adipocytes [28], while we saw no change in preadipocytes (Figure S1). These incongruencies may be due to differences in cell culture protocols. Regardless, we could not examine the reversibility of fibrotic phenotypes, and this should be investigated in future studies. Our observations regarding inflammatory cytokines were in the absence of changes to measures of differentiation, such as triglyceride content or expression of late adipogenic markers, indicating that cells were not undergoing de-differentiation, in agreement with the previously mentioned studies. The reduction in adiponectin and leptin may be due to several mechanisms, including but not limited to reduced production, impaired protein folding, or reduced release from the adipocyte [29]. Prior evidence has linked protease inhibitors to endoplasmic reticulum stress (ER stress) as a result of misfolded protein responses that are linked to adipocyte dysfunction [30]. While we found evidence of increased ER stress following exposure to both DTG and DRV (Figure 3), this was disconnected from the reduced adipokine production only seen in DTG-exposed cells. It is possible that the extent of ER stress conferred by the concentrations of drugs used was not sufficient to suppress adipokine production and that other pathways contributed to this decline.

Oxidative stress due to mitochondrial dysfunction is another established pathway that has been implicated in the development of dysfunctional adipocytes and impaired adiponectin secretion [31]. Others have indicated that INSTIs have adverse effects on mitochondria in CD4 T-cells [32]. Our study demonstrated that exposure to DTG but not DRV resulted in increased lactate production in both ASCs and newly differentiated cells, as well as suppressed basal and maximal mitochondrial oxygen consumption, indicative of impaired mitochondrial function. While we did not measure oxidative stress in this study, Gorwood et. al. demonstrated that adipocytes exposed to INSTIs had higher levels of oxidative stress related to mitochondrial dysfunction [28]. The mechanism by which integrase inhibitors affect mitochondrial function remains unclear; however, given calcium’s crucial role in mitochondrial function [33], INSTIs’ capacity to chelate calcium may be a contributing factor [34]. Others have shown DTG to suppress the expression of uncoupling protein 1 (UCP1) in thermogenic brown and beige adipocytes both in vivo and in vitro, leading to lower maximal oxygen consumption in these models [35]. While UCP1 expression is typically very low in the preadipocytes and white adipocytes used in this study, alterations in oxygen consumption may be due to alterations in overlapping adipogenic gene expression programs.

Our study indicated that INSTIs may have complex actions on the process of adipogenesis and clearly differ from the effects of DRV, a protease inhibitor. Cells exposed to DTG throughout differentiation had increases in PPARγ, the master regulator of adipogenesis, and adiponectin mRNA expression, but paradoxical suppression of adiponectin secretion and triglyceride accumulation. Others have found similar and competing results showing increased PPARγ and PPARγ target genes, and variable effects on triglyceride accumulation [9,21,22]. The regulation of PPARγ action is complex, involving many post-translational modifications, ligand binding, and cofactors that lend specificity to its actions [36]. It is possible that despite increased mRNA expression of PPARγ and adiponectin following exposure to INSTIs, there may be impairments in protein folding or other pathways related to cytokine production. The reduced triglyceride formation observed in cells exposed to DTG during differentiation could be explained by greater lipolytic activity; however, when free glycerol was measured, concentrations were not different when normalized to lipid content (Figure S4). Others have implicated INSTIs in the development of insulin resistance in adipocytes, which can negatively impact the differentiation of ASCs and lipid accumulation in these cells [9].

While others have shown the effects of DTG and other ART on ASCs and newly differentiated adipocytes, ours is the first to show that these effects are reversible at the level of the adipocyte. Although the data cannot comprehensively decipher the mechanism by which these ARTs alter adipocyte function, it allows for the identification of potentially toxic effects directly on these specific target cells. We were able to observe differential effects of an INSTI, DTG, from a PI, DRV, on ASC and newly differentiated adipocytes that may provide specific mechanisms that warrant further study. Importantly, the concentrations of drugs used in this study were within the physiological range (Cmax), and adipose cells isolated from different human donors were used for experiments, limiting the impact of variable susceptibility to the actions of these drugs.

We acknowledge there are limitations when applying these results to clinical practice. Our system isolates the effects of ART directly on adipocytes and precursors, but the potential impact of INSTIs on other cell populations relevant to metabolic homeostasis, such as hepatocytes, beta cells, and orexigenic neurons, could not be explored. Therefore, we cannot examine the in vivo interactions of these systems to fully understand the mechanisms of how INSTIs may drive weight gain or metabolic dysfunction in PWH. Recent data from the DEFINE study [15] may help in understanding the metabolic consequences of switching ART. Additionally, we only report on cells isolated from subcutaneous adipose depots. Visceral adiposity is more strongly linked to metabolic dysfunction, and stem cells and adipocytes isolated from these tissues can behave differently than the subcutaneous cells used in this study, indicating a need for examination of these tissue cell populations. Importantly, acquiring visceral cells is more invasive and difficult to isolate in humans, and the adipogenic capacity of subcutaneous cells is more strongly linked to metabolic parameters in humans [12]. For the purposes of this study, the inability of visceral ASCs to become adipocytes limited their use for experiments involving newly differentiated cells, as <10% of cells underwent adipogenesis. Finally, we examined only two ARTs from different drug classes, and each (DRV and DTG) in isolation. Most ART regimens are combinatorial in nature and certainly may confer different effects than single therapies. This is especially relevant given the ongoing examination of the relative effects of INSTIs versus their coformulation with tenofovir alafenamide, which has well-documented effects on weight gain [37,38]. Thus, it will be important in future studies to examine how different combinatorial ARTs may alter both adipocyte and mitochondrial function.

## 5. Conclusions

In conclusion, our study on the direct effects of an INSTI compared to a PI on adipocyte maturation and metabolism adds to the growing literature of the impact of INSTIs and ART on adipose tissue, a key organ in metabolic homeostasis. Importantly, the most consistent effect of INSTIs on newly differentiated adipocytes—the suppression of key adipokines, specifically adiponectin and leptin—was reversible when cells were switched to a protease inhibitor, DRV. While further data is necessary to confirm if the reversal of these phenotypes takes place in vivo, switching to equally effective medications may help halt or reverse negative metabolic consequences associated with INSTI exposure that may contribute to MUO in PWH.

## Data Availability

The original contributions presented in this study are included in the article/Supplementary Materials. Further inquiries can be directed to the corresponding author(s).

## Supplementary Materials

The following supporting information can be downloaded at: https://www.mdpi.com/article/10.3390/v18010149/s1, Figure S1: Proinflammatory and profibrotic markers in preadipocytes were unchanged after exposure to dolutegravir and darunavir; Figure S2: Exposure to Dolutegravir nor Darunavir did not alter adipocyte markers in newly differentiated adipocytes; Figure S3: Exposure to Dolutegravir nor Darunavir did not alter secretion of inflammatory cytokines in newly differentiated adipocytes; Figure S4: Exposure to Dolutegravir nor Darunavir did not alter basal lipolysis from newly differentiated adipocytes treated from differentiation; Table S1: ELISA Product Details; Table S2: Probes used for RT-qPCR.

## Abbreviations

The following abbreviations are used in this manuscript:

INSTI	Integrase Strand Transfer Inhibitor
PI	Protease Inhibitor
DTG	Dolutegravir
DRV	Darunavir
PWH	People With HIV
ASC	Adipose Stem Cells
MUO	Metabolically Unhealthy Obesity
ART	Antiretroviral Therapy
HIV	Human Immunodeficiency Virus
EFV	Efavirenz
CDM	Complete Differentiation Media
FBS	Fetal Bovine Serum
SDS	Sodium Dodecyl Sulfate
ELISA	Enzyme Linked Immunosorbent Assay
DMSO	Dimethylsulfoxide
IL-6	Interleukin-6
MCP-1	Monocyte Chemoattractant Protein-1
PPARγ	Peroxisome-proliferator-activated-receptor-gamma
LPL	Lipoprotein Lipase
FABP4	Fatty-acid binding protein 4
ANOVA	Analysis of Variance
ADIPOQ	Adiponectin
DDIT3	DNA Damage Inducible Transcript 3
ATF6	Activating Transcription Factor 6
ER	Endoplasmic Reticulum
INHBA	Inhibin Beta-A
ACTA2	Alpha-Actin
TIMP1	Tissue Inhibitor of Metalloproteinase 1
