# Supplementary material for: Reversible Effects of Integrase Inhibitors on Newly Differentiated Adipocytes
Source: Viruses. 2026 Jan 22;18(1):149. doi: 10.3390/v18010149 (PMC12846639; doi:10.3390/v18010149)
Supplement: Supplementary file 1 [file viruses-18-00149-s001.zip › viruses-4068214-supplementary.pdf]

Table S1. ELISA Product Details.

Target	Company	Product Name	Catalog Number	Detection range
IL-6	R&D Systems	**Human IL-6 ELISA Kit - Quantikine**	**D6050B**	3.1 - 300 pg/mL
MCP-1	R&D Systems	**Human CCL2/MCP-1 ELISA Kit - Quantikine**	**DCP00**	31.2 - 2,000 pg/mL
Adiponectin	R&D Systems	**Human Total Adiponectin/Acrp30 ELISA Kit - Quantikine**	**DRP300**	3.9-250ng/mL
Leptin	R&D Systems	**Human Leptin ELISA Kit - Quantikine**	**DLP00**	15.6-1000pg/mL

Table S2. Probes used for RT-qPCR

Probe	Reference number, Cat:4331182
INHBA	Hs01081598_m1
TIMP1	Hs01092511_m1
ACTA2	Hs00426835_g1
PPIA	Hs01565700_g1
ADIPOQ	Hs00605817_m1
PPARg	Hs01115513_m1
FABP4	Hs01086177_m1
ATF6	Hs00232586_m1
DDIT3	Hs10190850_m1

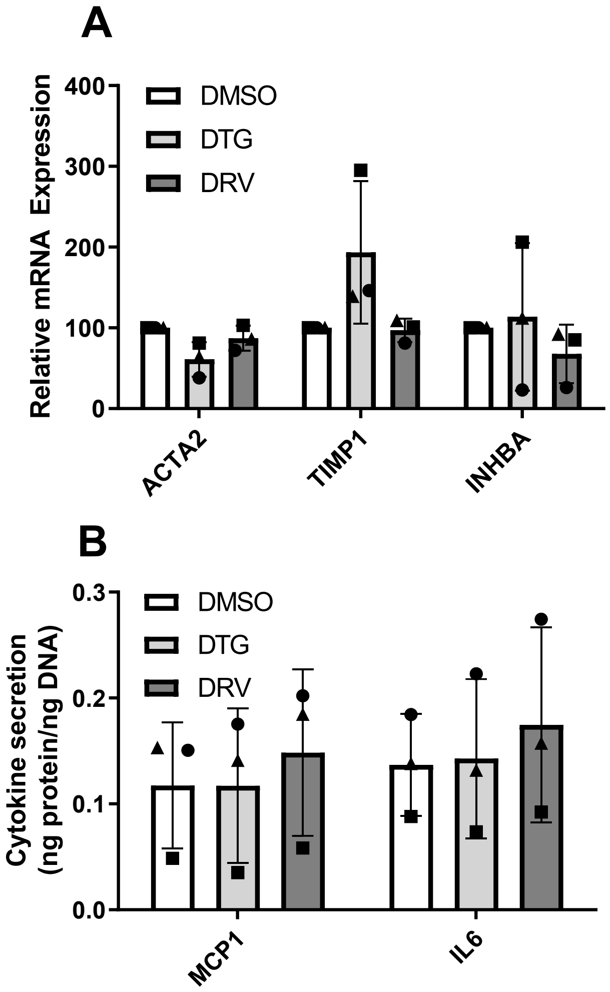

Figure S1. Proinflammatory and profibrotic markers in preadipocytes were unchanged after exposure to dolutegravir and darunavir. (A) mRNA expression profibrotic genes and (B) 24 hour media content of MCP1 and IL6 from ASCs exposed for 2 days to Dolutegravir (DTG), Darunavir (DRV), or control (DMSO).

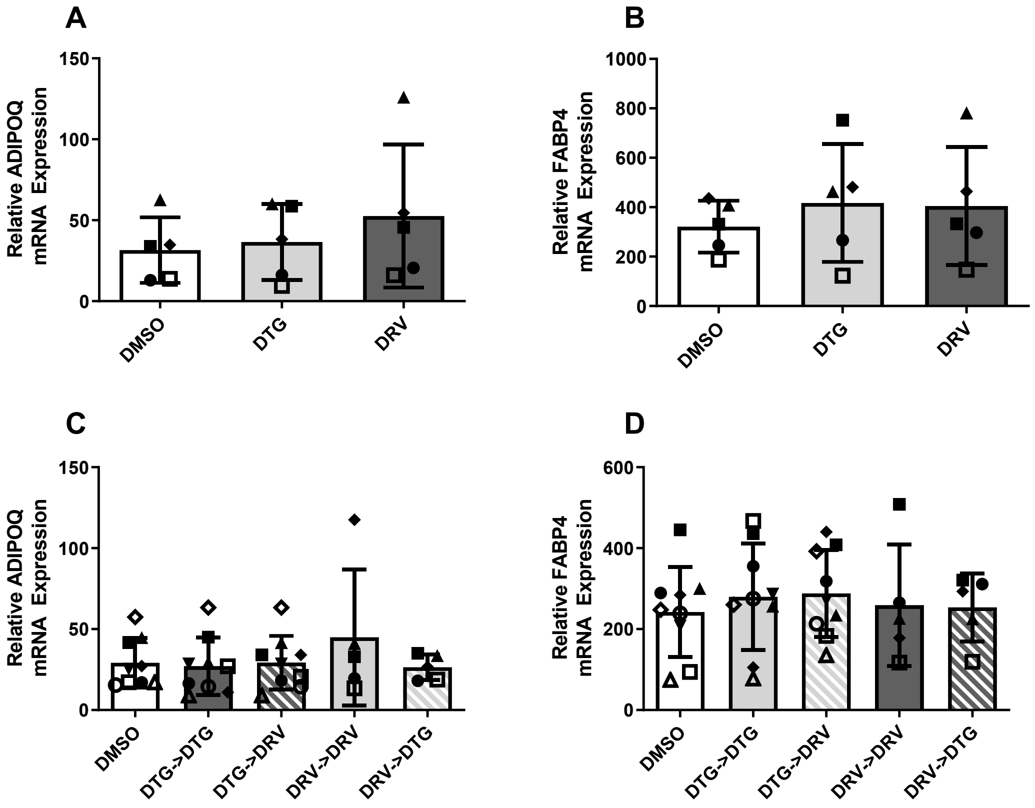

Figure S2. Exposure to Dolutegravir nor Darunavir did not alter adipocyte markers in newly differentiated adipocytes. mRNA expression of Adiponectin (A&C) and Fatty acid binding protein (B&D), late markers of adipogenesis, following 7 (A&B, n=5) or 14 (C&D, n=5-8) days of exposure to ARVs during maintenance; "->" indicates the switch at day 7 to the other drug. DMSO-Dimethylsulfoxide, DTG-Dolutegravir (3.1ug/mL), DRV-Darunavir (11.8uM)

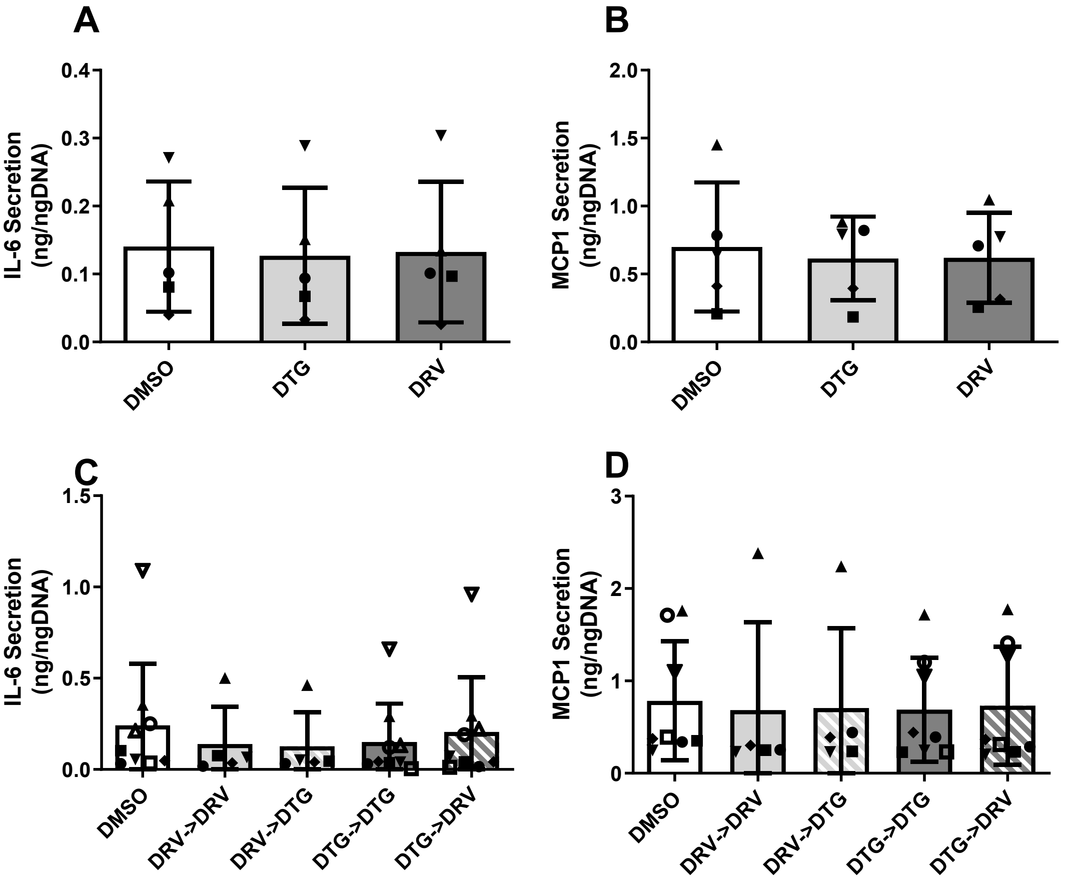

Figure S3. Exposure to Dolutegravir nor Darunavir did not alter secretion of inflammatory cytokines in newly differentiated adipocytes. Secretion of IL-6 (A&C), MCP-1 (B&D) after 7 (A&B) and 14 (C&D) days of exposure to DTG or DRV in newly differentiated adipocytes. "->" indicates a switch at day 7 to the indicated ARV.

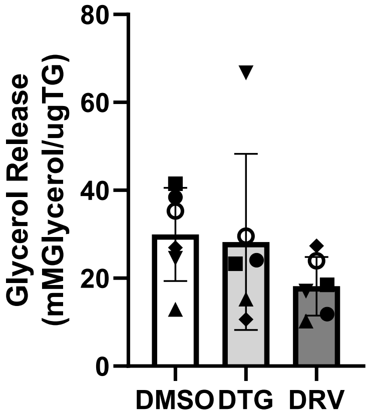

Figure S4. Exposure to Dolutegravir nor Darunavir did not alter basal lipolysis from newly differentiated adipocytes treated from differentiation. Glycerol concentrations in media from newly differentiated adipocytes as measured by enzymatic assay. n=6
